# Treatment strategies for relapse after CAR T-cell therapy in B cell lymphoma

**DOI:** 10.3389/fped.2023.1305657

**Published:** 2024-01-12

**Authors:** Shuto Negishi, James H. Girsch, Elizabeth L. Siegler, Evandro D. Bezerra, Kotaro Miyao, R. Leo Sakemura

**Affiliations:** ^1^ Department of Hematology and Oncology, Konan Kosei Hospital, Konan, Japan; ^2^ T Cell Engineering, Mayo Clinic, Rochester, MN, United States; ^3^ Division of Hematology, Mayo Clinic, Rochester, MN, United States; ^4^ Mayo Clinic Graduate School of Biomedical Sciences, Rochester, MN, United States; ^5^ Department of Molecular Medicine, Mayo Clinic, Rochester, MN, United States; ^6^ Department of Hematology and Oncology, Ohio State University, Columbus, OH, United States; ^7^ Department of Hematology and Oncology, Anjo Kosei Hospital, Anjo, Japan

**Keywords:** CAR T-cell therapy, salvage therapies, relapsed/refractory, B-cell lymphoma, real-world data

## Abstract

Clinical trials of anti-CD19 chimeric antigen receptor T (CART19) cell therapy have shown high overall response rates in patients with relapsed/refractory B-cell malignancies. CART19 cell therapy has been approved by the US Food and Drug Administration for patients who relapsed less than 12 months after initial therapy or who are refractory to first-line therapy. However, durable remission of CART19 cell therapy is still lacking, and 30%–60% of patients will eventually relapse after CART19 infusion. In general, the prognosis of patients who relapse after CART19 cell therapy is poor, and various strategies to treat this patient population have been investigated extensively. CART19 failures can be broadly categorized by the emergence of either CD19-positive or CD19-negative lymphoma cells. If CD19 expression is preserved on the lymphoma cells, a second infusion of CART19 cells or reactivation of previously infused CART19 cells with immune checkpoint inhibitors can be considered. When patients develop CD19-negative relapse, targeting different antigens (e.g., CD20 or CD22) with CAR T cells, investigational chemotherapies, or hematopoietic stem cell transplantation are potential treatment options. However, salvage therapies for relapsed large B-cell lymphoma after CART19 cell therapy have not been fully explored and are conducted based on clinicians' case-by-case decisions. In this review, we will focus on salvage therapies reported to date and discuss the management of relapsed/refractory large B-cell lymphomas after CART19 cell therapy.

## Introduction

Chimeric antigen receptor (CAR) T-cell therapy involves the genetic modification of autologous T lymphocytes to express a synthetic receptor. The typical CAR structure incorporates an antibody-derived single-chain variable fragment (scFv) in the extracellular domain, which is linked with a hinge domain and a transmembrane domain to anchor the receptor in the cell membrane. The scFv confers specificity to tumor-associated antigens (TAAs). After antigen recognition, CAR T-cell activation is initiated by an intracellular CD3ζ signaling domain, which is integral to the T-cell receptor (TCR)-CD3 complex and a primary activator of T-cell response. Second- and third-generation CAR T-cell signaling is augmented with one or more intracellular co-stimulatory domains, such as CD28 or 4-1BB (1). The inclusion of these co-stimulatory domains in subsequent generations of CAR constructs has been shown to enhance CAR T-cell antitumor efficacy (2) and persistence (3, 4). CD19-directed CAR T-cell (CART19) therapy has been approved for B-cell malignancies including B-cell precursor acute lymphoblastic leukemia (B-ALL), high-grade B-cell lymphoma, diffuse large B-cell lymphoma (DLBCL), primary mediastinal B-cell lymphoma (PMBCL), follicular lymphoma (FL), mantle cell lymphoma (MCL), and marginal zone lymphoma (5–14). In B-cell malignancies, particularly in relapsed or refractory DLBCL, response rates to CART19 cell therapy have been promising (15). In pivotal clinical trials, overall response rates (ORR) have often exceeded 50%, with many patients achieving complete remissions (CR). Due to these unprecedented outcomes, the U.S. Food and Drug Administration (FDA) has approved four different CART19 cell products for the treatment of B-cell malignancies, as detailed in Table 1. Although initial CAR T activity shows promise, resistance or relapse occurs in the majority of patients within 1–2 years (16, 17). The risk of disease relapse often depends on the depth of the initial response, the disease burden before therapy, and other factors like the presence of minimal residual disease (MRD) post-treatment. In this review, our main objectives are to (1) highlight the real-world data surrounding CAR T-cell therapy for B-cell lymphomas, (2) explore the mechanisms and challenges linked with resistance or failure of CAR T-cell therapy, and (3) discuss potential interventions for relapses following CAR T-cell therapy. Our aim is to provide insights that can direct both future research and clinical decision-making.

**Table 1:** FDA-approved CD19-directed CAR T-cell products.

CAR T-cell products	Brand name	Co-stimulatory domain	Indication	Trial data reported	ClinicalTrials.gov ID
Tisagenlecleucel	Kymriah	4-1BBζ	R/R pediatric and young adult (≤25 years) B-ALL	ELIANA	NCT02435849
			R/R adult DLBCL, transformed FL	JULIET	NCT02445248
			R/R FL	ELARA	NCT03568461
Axicabtagene ciloleucel	Yescarta	CD28ζ	R/R adult DLBCL, HGBL, transformed FL	ZUMA-1	NCT02348216
			R/R FL	ZUMA-5	NCT03105336
Lisocabtagene maraleucel	Breyanzi	4-1BBζ	R/R adult DLBCL, HGBL, transformed FL, PMBCL, FL3B	TRANSCEND NHL 001	NCT02631044
Brexucabtagene autoleucel	Tecartus	CD28ζ	R/R MCL	ZUMA-2	NCT02601313
			R/R adult B-ALL	ZUMA-3	NCT02614066

R/R, relapsed or refractory; B-ALL, B-cell acute lymphoblastic leukemia; DLBCL, diffuse large B-cell lymphoma; FL, follicular lymphoma; HGBL, high-grade B-cell lymphoma; PMBCL, primary mediastinal B-cell lymphoma; FL3B, follicular lymphoma grade 3B; MCL, mantle cell lymphoma.

## Materials and methods

We conducted a systematic review of reports on large B-cell lymphoma (LBCL), using PubMed and adhering to the Preferred Reporting Items for Systematic Reviews and Meta-Analyses (PRISMA) guidelines (18). Our key search terms were “CAR T-cell therapy and real-world data” and “CAR T-cell therapy and B-cell lymphoma”. We also searched for relevant studies in the reference lists of the included articles. Our focus was on studies involving patients with LBCL who received CART19 cell therapy. We excluded case reports and clinical trials. From these reports, we extracted data on the countries, number of patients, types of CAR T-cell products used, ORR, CR rate, 1-year overall survival (OS), 1-year progression-free survival (PFS), and the incidence of cytokine release syndrome (CRS) and immune effector cell-associated neurotoxicity syndrome (ICANS). Disease responses were evaluated using the International Working Group (IWG) revised response criteria for malignant lymphoma and the Lugano Classification (19). For adverse events, CRS was assessed using either the Lee criteria or the CAR T-cell Therapy Associated Toxicity (CARTOX) criteria (20). ICANS was graded according to the CARTOX criteria, the Common Terminology Criteria for Adverse Events (CTCAE), or the American Society for Transplantation and Cellular Therapy (ASTCT) consensus criteria (21).

## Real-world data of CD19-directed CAR T-cell therapy in patients with B-cell lymphoma

### Large B-cell lymphoma (LBCL)

According to the ZUMA-1 pivotal study, the ORR, 1-year OS rate, and 1-year PFS of axicabtagene ciloleucel (axi-cel) against LBCL were 82%, 62%, and 45%, respectively (22). The JULIET pivotal clinical trial for tisagenlecleucel (tisa-cel) showed ORR, 1-year OS, and 1-year PFS of 52%, 45%, and 35%, respectively (23). To assess real-world data, we extracted data from 27 studies that analyzed the outcomes of CAR T-cell therapy in real-world settings, following the methods outlined above. In summary, an analysis of real-world data from these studies revealed that among patients with LBCL undergoing CART19 cell therapy, the median ORR was 67% (range: 46%–82%), the CR rate was 49% (range: 30%–80%), the 1-year OS rate was 56% (range: 45%–83%), and the 1-year PFS rate was 36% (range: 27%–64%) (summarized in Table 2, detailed in Table 3). Eight studies specifically focused on treatment outcomes with axi-cel, demonstrating ranges for the ORR of 68%–82%, the 1-year OS of 49%–83%, and the 1-year PFS of 37%–64% (24–31). Four studies focused on tisa-cel, showing treatment outcomes with an ORR of 46%–62%, a 1-year OS of 45%–58%, and a 1-year PFS of 27%–55% (32–35). Although directly contrasting real-world data with results from pivotal clinical trials poses challenges, the outcomes appear to be comparable. Nevertheless, it is important to note that the majority of these data originates from Europe and the U.S.; incorporating Asian datasets would further enrich the analysis and provide a more comprehensive overview.

**Table 2:** Summary of real-world data.

ORR (%, median, range)	CR (%, median, range)	1-year OS (%, median, range)	1-year PFS (%, median, range)
67 (46–82)	49 (30–80)	56 (45–83)	36 (27–64)

ORR, Overall response rate; CR, Complete response; OS, Overall survival; PFS, Progression-free survival.

**Table 3:** Summary of real-world data.

Author	Country	Patient number	Axi-cel		Tisa-cel		ORR (%)	CR (%)	1-year OS (%)	1-year PFS (%)	CRS All Grade (%)	CRS > Grade 3 (%)	ICANS All Grade (%)	ICANS > Grade 3 (%)
Bethge	Germany	356	173	(49%)	183	(51%)	65	37	52	30	73	12	33	11
Ghafouri	US	53	45	(85%)	8	(15%)	72	64	55	45	68	6	30	19
Yagi	Japan	21	0	(0%)	21	(100%)	NA	62	58	55	86	10	5	0
Rejeski	Germany	106	68	(64%)	38	(36%)	66	44	55	29	92	9	47	19
Abbasi	US	10	10	(100%)	0	(0%)	NA	80	NA	NA	60	NA	50	NA
Rabinovich	US	31	31	(100%)	0	(0%)	71	65	NA	NA	100	NA	100	NA
Ayuk	Germany	21	21	(100%)	0	(0%)	NA	NA	49	37	71	14	48	19
Stolz	Switzerland	21	17	(81%)	4	(19%)	62	38	61	30	74	13	30	9
Bastos-Oreiro	Spain	192	101	(53%)	91	(47%)	60	36	50	35	78	6	29	11
Penack	Europe	398	246	(62%)	152	(38%)	62	38	56	33	NA	NA	NA	NA
Casadei	Italy	30	18	(60%)	12	(40%)	77	50	70	38	87	10	43.3	16.6
Bachy	France	418	209	(50%)	209	(50%)	77	50	59	41	NA	NA	NA	NA
Sesques	France	61	28	(46%)	33	(54%)	63	48	50	35	85	8	28	10
Iacoboni	Spain	75	0	(0%)	75	(100%)	60	32	45	32	71	5	15	1
Vercellino	France	116	67	(58%)	49	(42%)	NA	NA	67	47	NA	NA	NA	NA
Nastoupil	US	275	275	(100%)	0	(0%)	82	64	68	47	91	7	68.7	31
Jacobson	US	1,297	1,297	(100%)	0	(0%)	73	56	62	47	83	14	55	34
Jacobson	US	122	122	(100%)	0	(0%)	70	50	67	NA	93	16	70	35
Cuffel	France	30	0	(0%)	30	(100%)	46	30	53	27	NA	NA	NA	NA
Iovino	US	60	60	(100%)	0	(0%)	68	32	NA	NA	77	13	52	32
Pasquini	US	155	0	(0%)	155	(100%)	62	40	55	28	45	5	18	5
Crombie	US	33	33	(100%)	0	(0%)	76	67	83	64	88	6	39	27
Sermer	US	69	46	(67%)	23	(33%)	72	52	64	44	NA	NA	NA	NA
Kwon	Spain	261	133	(51%)	128	(49%)	57	38	48	34	81	7	30	11

Axi-cel, axicabtagene ciloleucel; Tisa-cel, tisagenlecleucel; ORR, overall response rate; CR, complete response; OS, overall survival; PFS, progression-free survival; CRS, cytokine release syndrome; ICANS, immune effector cell-associated neurotoxicity syndrome; US, United States; NA, not applicable.

Several retrospective studies have focused on identifying the risk factors that worsen the outcomes of CAR T-cell therapy. Nastoupil et al. studied 275 LBCL patients treated with axi-cel. Their multivariate analysis identified factors which negatively impacted OS, including elevated serum total bilirubin (≥1.5 g/dl) and lactate dehydrogenase (LDH) levels at the time of axi-cel administration, male gender, Eastern Cooperative Oncology Group (ECOG) Performance Status (PS) > 2, undergoing bridging therapies, and primary refractory status. Similarly, elevated total bilirubin and LDH levels at the time of axi-cel administration, male gender, ECOG PS > 2, and age below 60 were identified as risk factors for low PFS (27). The finding that high total serum bilirubin count correlated with worse PFS and OS was notable. Given that high serum LDH levels also negatively impacted outcomes post-CAR T-cell therapy, we speculated that elevated bilirubin counts also reflected a high tumor burden at the time of CAR T-cell therapy. Bethge et al. evaluated 356 patients who received either axi-cel or tisa-cel for LBCL. Their findings indicated that non-response to bridging therapies, high LDH levels, and a worse ECOG PS were negative factors for both OS and PFS. Moreover, they found that older age and tisa-cel treatment correlated with poorer PFS outcomes (36). While more research is needed, the disease status at the time of CAR T-cell administration and the performance status of patients are potentially significant predictors of OS and PFS outcomes.

### Mantle cell lymphoma (MCL) and follicular lymphoma (FL)

Brexucabtagene autoleucel (brexu-cel) is FDA-approved for the treatment for MCL (37). For FL, both axi-cel and tisa-cel therapies have gained FDA approval (38). In this section, we focus on these three CAR T-cell products as novel treatment options for MCL and FL (Table 1).

The ZUMA-2 study validated the efficacy of CART19 for MCL (39). In this phase II trial, 68 patients with MCL who had relapsed or were refractory to conventional therapies were treated with brexu-cel. These patients had previously undergone 1 to 5 different treatment regimens. The observed ORR was 91%, with a CR of 68%. At the 1-year mark, survival rates were 80% for OS and 62% for PFS (39). Three separate studies evaluated real-world data of brexu-cel for MCL. The study led by Wang et al. of 167 MCL patients provided the most extensive dataset. They reported an ORR of 89%, and 6-month OS and PFS rates of 85% and 63%, respectively (37). The occurrence of adverse events in real-world MCL data closely reflected those reported in the ZUMA-2 study.

The ZUMA-5 study assessed the effectiveness of axi-cel in patients with indolent lymphomas, 83% of whom had FL; in 153 patients with FL or marginal zone lymphoma that was refractory or relapsed after at least two lines of treatment, the ORR was 95%. The 1-year OS and PFS stood at 80% and 78%, respectively (14). The ELARA study, a phase II trial, evaluated tisa-cel in 97 patients with relapsed/refractory FL and reported an ORR of 86% (40). While these studies highlight the potential of CAR T-cell therapy in patients with FL, real-world data remain sparse. Given the recent approval of CART19 cell therapy for FL, continuous monitoring of real-world scenarios is crucial.

## Post-CAR T-cell therapy tumor relapse: mechanisms and salvage therapies

Despite the encouraging outcomes of CAR T-cell therapy in both pivotal clinical trials and real-world scenarios, 30 to 60% of patients relapse after CAR T-cell therapy, and 10 to 20% of relapsed patients experience CD19-negative relapse (41). When tumors do not respond to CAR T-cell therapy, therapeutic options become extremely limited. For example, in patients with DLBCL who have relapsed or are refractory after CAR T-cell therapy, the only remaining treatment option with curative potential is an allogeneic hematopoietic stem cell transplant (HSCT) (38). Additionally, there is a lack of established salvage therapies for such cases, often resulting in a poor prognosis.

**Figure 1:**
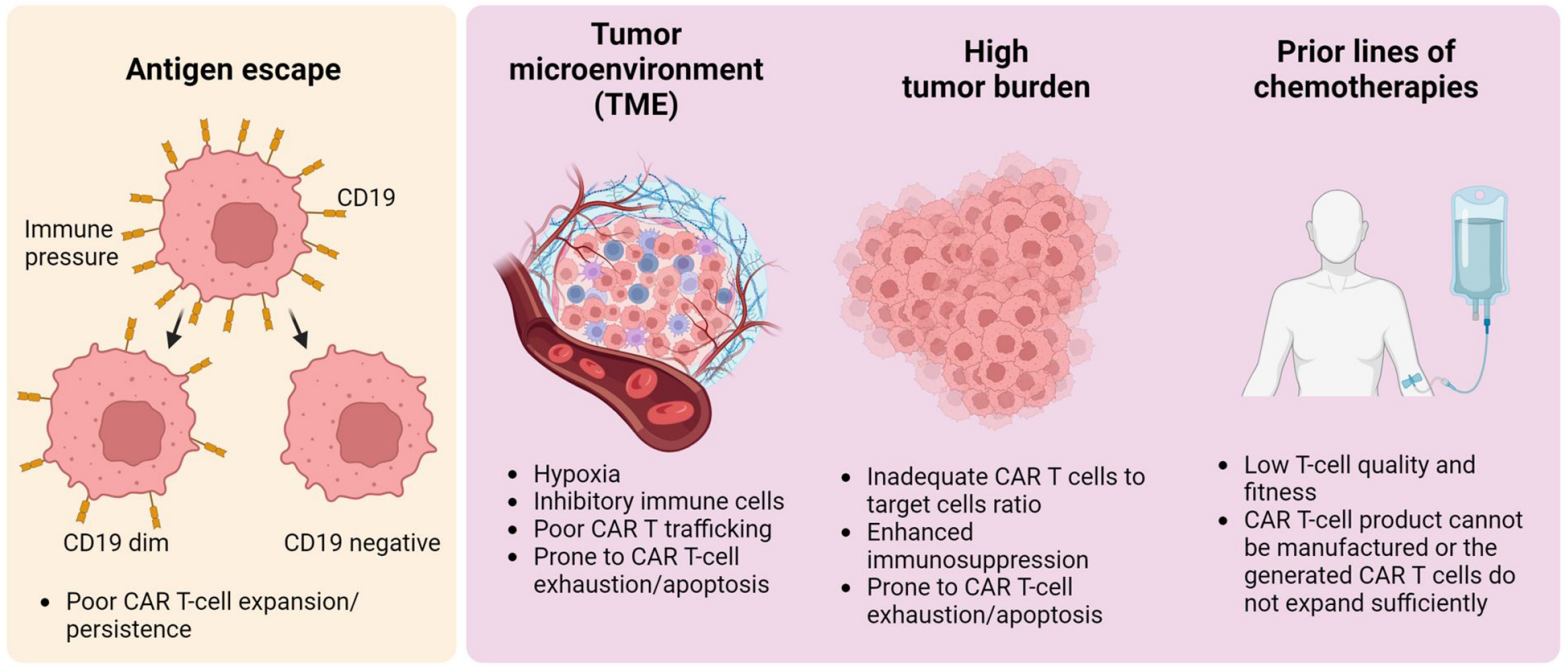
General overview of tumor relapse post-CAR T-cell therapy. Multiple factors contribute to CAR T-cell therapy failure. The left panel illustrates antigen modulation — specifically, the reduction or loss of CD19 on malignant B-cells — which leads to antigen escape. This escape mechanism fosters resistance to CAR T-cell therapy, a phenomenon not limited to B-cell malignancies but also occurring in other types, such as solid tumors. The right panel shows CAR T-cell dysfunction, attributed to the tumor microenvironment, high tumor burden, or intrinsic T-cell defects resulting from prior chemotherapy treatments. These elements result in inadequate CAR T-cell expansion or lead to CAR T-cell apoptosis/exhaustion *in vivo*, potentially causing lymphoma recurrence.

It is worth noting that manufacturing issues during CAR T-cell production play a role in subsequent CAR T-cell failure due to low transduction efficiency, insufficient CAR T-cell expansion *ex vivo*, or low CAR T-cell viability after thawing (41, 42). Beyond problems with CAR T-cell manufacturing, multiple primary mechanisms contributing to resistance in CAR T-cell therapy have been identified: antigen escape, modulation of the tumor microenvironment (TME), substantial tumor burden upon initiation of CAR T-cell therapy, and exposure to prior cytotoxic treatments for lymphoma (Figure 1). These elements collectively contribute to the eventual failure of CAR T-cell therapy, predominantly due to the induction of CAR T-cell exhaustion or apoptosis, reflecting complex interactions at the cellular and molecular levels. Here, we address each mechanism that leads to tumor relapse and resistance following CAR T-cell therapy.

### Antigen escape

One primary mechanism linked to tumor resistance or relapse following CAR T-cell therapy is the total or partial loss of the target antigen or mutations in the epitope recognized by the CAR. This can be orchestrated by malignant B cells through several genetic modifications. These include hemizygous deletion of the CD19 locus, leading to a permanent loss of CD19 expression on the cell surface; mutations causing alternative splicing of CD19 mRNA, which can alter the molecular structure of CD19; and epigenetic changes resulting in CD19 gene silencing (43, 44). In the ZUMA-1 trial, CAR T-cell therapy failed in eleven patients. Evaluation of lymphoma tissues from these patients revealed CD19-negative lymphoma cells in three of eleven cases (27%). Moreover, CAR-T cell therapy might also induce a lineage shift in malignant B cells, changing them to a myeloid phenotype through the downregulation of key B-cell transcription factors including Pax5 (44).

### Tumor microenvironment (TME)

The lack of durable responses after CAR T-cell therapy has been widely attributed to the immunosuppressive TME. This can lead to insufficient expansion and infiltration of CAR T cells and ultimately, treatment failure. Within the TME, various cells such as myeloid-derived suppressor cells (MDSCs), regulatory T-cells, and tumor-associated macrophages (TAMs) contribute to immune suppression (45). The interaction of programmed cell death protein-1 (PD-1) on tumor-specific T cells with programmed cell death-ligand 1 (PD-L1) or PD-L2 on TAMs, MDSCs, or cancer-associated fibroblasts in the TME prompts cell death and reduces the number of cytotoxic T cells in the tumor tissue (46). There is increasing evidence to suggest that limited expansion of CAR T-cells and reduced durable response rates are linked to increased interferon signaling, which upregulates PD-L1 on tumor cells in the TME and on circulating MDSCs (47). The abundance of suppressive cytokines such as interleukin (IL)-4, IL-13, transforming growth factor (TGF)-β, IL-6, and IL-10 in the TME can further inhibit CAR T-cell function (46, 48, 49).

### High tumor burden

Findings from the American CAR T-Cell Consortium suggest that elevated LDH levels prior to CAR T-cell therapy are linked to poorer survival outcomes (50). In addition, data from the ZUMA-1 study suggest that a larger initial tumor burden decreases the likelihood of sustained therapeutic responses (51). Analysis from the JULIET trial highlighted that patients with higher LDH levels before infusion, severe thrombocytopenia before infusion, and severe ICANS experienced poorer PFS and OS (52). A study from France also highlighted that LBCL patients with extensive disease with multiple extranodal lesions were at an increased risk of disease relapse post-CAR T-cell treatment, including axi-cel and tisa-cel (53). In a separate study of 273 relapsed or refractory patients with LBCL treated with CART19 across two centers. Further analysis from this research underscored that individuals with substantial disease burden at the time of apheresis exhibited a decreased OS rate (54). A high tumor burden generally results in an inadequate ratio of CAR T cells to target cells, increased complexity in the TME, and consequently, a greater tendency for CAR T cells to undergo exhaustion and apoptosis.

### Prior treatment

Prior lines of cytotoxic treatment lead to lymphopenia, preventing adequate collection of T cells for CAR T-cell therapy (55). Moreover, prior chemotherapy perturbs the *in vivo* metabolic functions of T cells, potentially undermining the long-term reactive capacity of CAR T cells (56). Clinical investigations have revealed that administration of agents like cytarabine and cyclophosphamide reduces the presence of early T-cell phenotypic subsets, and variations in these T-cell subsets correlate with the expansion potential of CAR T-cells (55).

### CAR T-cell exhaustion

T-cell exhaustion, which is characterized by a reduction in T-cell proliferation and effector functions, can arise from prolonged antigen stimulation. CAR T-cell exhaustion results in reduced therapeutic efficacy (57, 58) and is associated with resistance to treatment. Studies have shown that a larger population of exhausted CD8^+^ T-cells in the apheresis lymphocyte sample can be linked to relapsed/refractory outcomes following CAR T-cell treatments (29, 59). Notably, high levels of lymphocyte-activation gene-3 (LAG-3) and T-cell immunoglobulin and mucin domain-containing protein 3 (TIM-3) in CAR T-cell infusion samples are associated with reduced ORR of CART19 cell therapy and an increased likelihood of early DLBCL relapse (59). Furthermore, a rise in T-cells expressing LAG-3, paired with a diminished capacity to release cytokines upon activation, can reduce the efficacy of CART19, resulting in relapse with CD19-positive cells (41).

### Apoptosis of CAR T cells

Apoptosis is a well-defined cellular process leading to programmed cell death. In order to maintain the homeostatic balance between cell death and cell proliferation, apoptosis regulates cell fate and survival (60). In the context of CAR T cells, excessive CAR T-cell apoptosis can significantly reduce therapeutic efficacy. Multiple factors can induce CAR T-cell apoptosis, including activation-induced cell death (AICD) post-antigen recognition, negative feedback from regulatory cells or cytokines, or challenges posed by the TME, such as hypoxia or nutrient deprivation (61). It is worth noting that while intrinsic defects can render CAR T cells more susceptible to apoptosis, external factors play a considerable role in modulating this cell death pathway. Ghosh et al. demonstrated that overexpressed proapoptotic proteins (e.g., Bim, Bid, and FasL) in CAR T-cells are associated with T-cell differentiation and loss of self-renewal ability. They also showed that potent and cumulative T-cell stimulation leads to functional exhaustion and apoptosis of CAR T cells (62).

## Managing tumor relapse after CAR T-cell failure

In the ZUMA-1 trial of axi-cel, of the 52 non-responders to initial CAR T-cell therapy, nine were re-administered with axi-cel (40). Among these, five showed some clinical response: two achieved CR, and three had PR but ultimately relapsed. In the previously mentioned study by Tomas et al., they examined 182 patients with LBCL who had either relapsed or become refractory after CAR T-cell therapy. Of this cohort, 135 patients underwent various salvage treatments, including lenalidomide-based regimens, anthracycline and platinum drug-based therapies, polatuzumab vedotin (an antibody–drug conjugate consisting of a monoclonal antibody against CD79b and the anti-mitotic cytotoxic agent monomethyl auristatin)-based regimens, Bruton's tyrosine kinase inhibitor (BTKi)-based treatments, immune checkpoint inhibitor (ICI, specifically a monoclonal antibody against PD-1)-based therapies, radiation-based approaches, and other treatment agents from clinical trials. The lenalidomide group predominantly consisted of older patients who had been subjected to more treatment regimens prior to receiving CAR T-cell therapy. Patients in the anthracycline and platinum drug group often exhibited advanced disease stages and had previously shown resistance both to prior lines of treatment and the CAR T-cell therapy itself. Notably, those treated with polatuzumab vedotin and BTKi regimens had a median age below 60 and were more likely to have elevated LDH levels at diagnosis. In terms of treatment outcomes, the ORR and CR across all patients were found to be 39% and 20%, respectively. Furthermore, the one-year OS and PFS rates following CAR T relapse stood at 30% and 7.5%. Specifically, the ORRs for each treatment group were 33% for lenalidomide, 41% for anthracycline and platinum drugs, 48% for polatuzumab vedotin, 43% for BTKi, 20% for ICI, 54% for radiation, and 28% for clinical trials. The one-year OS was 69% for the lenalidomide group, 25% for the anthracycline and platinum drug group, 37% for the polatuzumab vedotin group, and 43% for the BTKi group. Remarkably, there were no significant differences in treatment efficacy across these groups. A multivariate analysis offered further insights, suggesting that patients over the age of 65 and those with elevated LDH levels at relapse had shorter OS. However, the use of lenalidomide seemed to enhance OS rates (54).

In a study by Di Blasi et al., 238 patients with aggressive B-cell lymphoma who had relapsed or become refractory after CAR T-cell treatment were evaluated (63). Of these, 57% had been treated with axi-cel, while 42.9% received tisa-cel. The one-year OS and PFS rates for these patients were 27% and 18%, respectively, highlighting the bleak outcomes after CAR T-cell therapy failures. Of these 238 patients, 154 received various salvage treatments. The therapeutic modalities administered were as follows: 38% received lenalidomide, 7% received a bispecific antibody targeting CD20 and CD3, 21% received molecular targeted therapies (among whom 73% underwent monotherapy and 27% underwent combination therapy), 11% received radiation therapy, 20% received immunochemotherapy (including rituximab-dexamethasone, high-dose Ara-C, oxaliplatin; rituximab-ifosfamide, carboplatin, etoposide; and polatuzumab vedotin-rituximab-bendamustine), and 0.6% received corticosteroids. Notably, radiation therapy was reserved only for patients with distinct lesions. Among the 120 patients available for assessment, the ORR was just 14%. The ORRs for individual treatments were 11% for lenalidomide, 14% for bispecific antibody, 11.5% for molecular target drugs, 36% for radiation, and 8% for immunochemotherapy. Median OS periods for each treatment group were 7.5 months for lenalidomide, 8.5 months for bispecific antibody, 4.5 months for molecular target drugs, 9.6 months for radiation, and 3.7 months for immunochemotherapy, showing varied results. Median PFS periods varied slightly, with 3.8 months for lenalidomide, 3.7 months for bispecific antibody, 2.1 months for molecular target drugs, 3.7 months for radiation, and 2.4 months for immunochemotherapy. Interestingly, there were no significant differences in ORR, median OS, or PFS across these treatment groups (63).

Further analysis revealed that patients with elevated LDH and ferritin levels prior to CAR T-cell therapy were more likely to have shorter PFS (63). Factors contributing to shortened OS included increased LDH and C-reactive protein levels as well as relapse or resistance observed before day 30 post-CAR T-cell treatment. Consistent with the study by Tomas et al., lenalidomide treatment was associated with improved OS (63).

**Figure 2:**
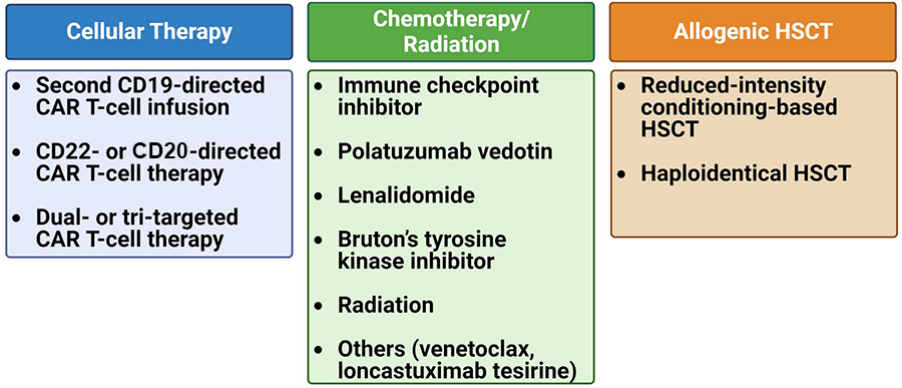
Treatment options for relapsed/refractory B-cell lymphoma post-CAR T-cell therapy. The treatment strategy upon disease recurrence can be categorized into three main groups: cellular-based therapy, chemotherapy/radiation therapy, and allogeneic hematopoietic stem cell transplantation (HSCT).

In conclusion, both studies underscore the challenging prognosis faced by patients with relapsed/refractory LBCL post-CAR T-cell therapy and the limited efficacy of current salvage regimens. Below, we discuss these potential agents as well as cellular therapy approaches that can be employed as salvage therapies for relapse post-CAR T-cell therapy (Figure 2).

### Lenalidomide

In the studies we previously discussed, lenalidomide was administered to 5%–38% of patients post-CAR T-cell therapy, indicating it as a moderately used salvage treatment following CAR T-cell administration (54, 63). Distinct advantages of lenalidomide include its oral administration route and a comparatively lower risk of side effects than alternative treatments. Some studies even highlighted higher OS and ORR compared to standard chemotherapies (54, 63). In specific cases, lenalidomide has been combined with other treatments. For instance, obinutuzumab, a recombinant type II anti-CD20 and immunoglobulin G1 Fc-optimized monoclonal antibody, was paired with lenalidomide for a patient who relapsed post-axi-cel therapy, leading to CR after five cycles of treatment (64). There is also an emerging concept of using lenalidomide as a maintenance therapy post-CAR-T. A study by Ping et al. assessed 16 patients with DLBCL treated with CAR T-cell therapy (62). Of these patients, seven received lenalidomide maintenance; starting 15 days post-CAR T-cell infusion, lenalidomide was given for 21 days, followed by a 7-day rest period. The remaining nine patients did not receive lenalidomide maintenance. While the ORR was similar in both groups, the 1-year OS rate was notably higher in the lenalidomide group, at 100% compared to 33% (65). The study suggests that lenalidomide might ameliorate CAR T-cell exhaustion (65). Compared to other agents, lenalidomide showed improved ORR, OS, and PFS in several studies (54, 63). Further preclinical and clinical analyses are expected to elucidate the interactions between lenalidomide and CAR T cells.

### Polatuzumab vedotin

Polatuzumab vedotin is an antibody–drug conjugate composed of a monoclonal antibody against CD79b and the anti-mitotic cytotoxic agent monomethyl auristatin. This biological drug is a therapeutic agent used as a salvage therapy following CAR T-cell treatment and has been frequently cited in various studies concerning salvage regimens, as mentioned earlier (42, 52). Gouni et al. specifically explored patients with LBCL who relapsed or developed resistance after CAR T-cell therapy and subsequently underwent polatuzumab vedotin treatment (60). In this study, 57 patients were administered polatuzumab vedotin, and 61% of these patients also received bendamustine, while a significant 95% were also given rituximab. The ORR was 44%; the ORR for patients who did or did not receive bendamustine was 55% and 38%, respectively, showing similar outcomes. At the 6-month mark, the OS rate was 40%, and the PFS rate was 20%. Advanced analysis identified bone marrow invasion and elevated LDH levels as potential risk factors for low PFS. While a relatively small number of patients has been treated with polatuzumab vedotin combination therapies, it is essential to acknowledge that the effectiveness of these treatments remains somewhat limited (66). Based on these data, it is still too early to conclude that polatuzumab vedotin is a beneficial salvage option for patients with relapsed LBCL following CAR T-cell therapy.

### Immune checkpoint inhibitor (ICI)

In small patient groups with aggressive B-cell lymphomas who relapsed after CAR T-cell therapy, results with ICI therapy using anti-PD-1 antibodies have been inconsistent. Major et al. conducted a comprehensive retrospective study to assess the efficacy of ICI therapy in these cases, examining the clinical data of 96 patients from 15 US academic centers. Many of these patients were diagnosed with DLBCL (53%), treated with axi-cel (53%), showed early relapse within 180 days post-CAR T (83%), and subsequently received either pembrolizumab (49%) or nivolumab (43%). ICI therapy yielded an ORR of 19% and a CR rate of 10%, with a median response duration of 221 days. Median values for PFS and median OS were 54 and 159 days, respectively. Those diagnosed with PMBCL had comparatively better outcomes with ICI therapy. Moreover, for patients with a later relapse post-CAR T (>180 days), both PFS and median OS were significantly higher than in those who relapsed earlier (128 vs. 51 days for PFS and 387 vs. 131 days for median OS). Adverse events of Grade ≥3 were reported in 19% of those on ICI therapy. Unfortunately, only 5% had durable response to ICI. From this expansive patient cohort study, ICI therapy seems less promising for aggressive B-cell lymphoma patients relapsing post-CAR T-cell treatment, particularly for those with early relapses. Therefore, ICI does not appear to be an optimal salvage treatment following CAR T-cell therapy (67).

### Radiation therapy

Radiation therapy (RT) has been documented to offer therapeutic benefits for patients with relapsed or refractory status post-CAR T-cell therapy. Ababneh et al. studied 120 patients undergoing salvage therapies post-CAR T-cell treatment (63). The initial salvage interventions comprised of RT for 25 patients, systemic therapy for 80, and a combination of these treatments for 15. The patient cohort was divided between those treated with axi-cel (56%) and tisa-cel (44%). The median duration of OS remained undefined for the RT group, was 6.6 months for the systemic therapy group, and 7.3 months for those receiving both treatments. The median period for PFS was 3.5 months for the RT group, 1.9 months for the systemic therapy group, and 3.3 months for the combination group. The outcomes were statistically similar across these groups. Of note, within the systemic therapy cohort, some patients transitioned to RT after the systemic approaches proved ineffective. Altogether, 54 patients (with a total of 93 lesions) underwent RT. From this subset, the median radiation dose was 30 Gy (range: 4–50 Gy), and the typical number of sessions was 10 (range: 1–28). In terms of technique, 64% received three-dimensional conformal radiation therapy (3D-CRT), 25% underwent intensity modulated radiation therapy (IMRT), 7% experienced both, and 4% were treated with an electron beam. Of the 81 assessable lesions, the ORR and CR were 82% and 59%, respectively, with a 1-year PFS rate of 84% (68).

Another study by Imber et al. assessed 14 patients given RT post-CAR T-cell therapy (64). Of these patients, 86% had DLBCL, 7% had high-grade BCL, and 7% had MCL. A majority (71%) had RT as their primary salvage intervention. The techniques were distributed as 50% receiving IMRT, 36% undergoing conventional photon RT, and 14% being treated with 3D-CRT. The typical radiation dose was 30 Gy (range: 20–46 Gy) over a median of 13 sessions (range: 5–30). Additionally, five patients had chemotherapy concurrently with RT, while three had chemotherapy subsequently. Of the 13 evaluable patients, those with confined lesions (6 patients) had an ORR of 100% and a CR of 50%. Conversely, the seven patients in progressive stages had an ORR of 71% but had no CR. The median OS for all patients was 10 months. In summation, while RT appears to offer outcomes on par with or superior to chemotherapy, direct comparisons are challenging since RT is generally preferred for patients with fewer lesions and a favorable ECOG PS score (69).

### Second CAR T-cell infusion

Gauthier et al. have explored the efficacy of a second dose of CART19 cells (CART2) as a potential means to enhance treatment results. In their phase I/II study (NCT01865617), they assessed 44 patients with B-cell malignancies which were relapsed/refractory after an initial dose of CART19 cells (CART1). CART1 was administered in three different dosages at 2 × 10^5^/kg, 2 × 10^6^/kg, or 2 × 10^7^/kg in 14 patients with B-ALL, 9 patients with chronic lymphocytic leukemia (CLL), and 21 patients with non-Hodgkin lymphoma (NHL). Even though 82% of these patients had their CART2 dose (either 2 × 10^6^/kg or 2 × 10^7^/kg) increased from their CART1 dose, severe adverse events were minimal: only 9% experienced grade ≥3 CRS, and 11% had grade ≥3 neurotoxicity. Post-CART2 treatment resulted in CR in 22% of CLL, 19% of NHL, and 21% of ALL cases. Median response durations were 33 months for CLL, 6 months for NHL, and 4 months for ALL following CART2. They highlighted that adding fludarabine to a cyclophosphamide preconditioning regimen before CART1 treatment, along with a dose increase for CART2 compared to CART1, were factors that contributed significantly to better response rates and prolonged PFS after CART2 (70).

While these studies showed that a second round of CART19 cell treatment can be effective, its success can be hampered by mutations or loss of the CD19 antigen. Even though the re-administration of CART19 cells demonstrated some efficacy in initial non-responders, there is an urgent need to tackle relapses that exhibit CD19-negative variations. Furthermore, one critical consideration is the potential immunogenicity elicited by the murine-derived FMC63 single chain variable fragment utilized in FDA-approved CART19 constructs, which risks compromising subsequent re-infusion approaches (22, 71).

### CD22- or CD20-directed CAR T-cell therapy

The rise of antigen-negative malignant B-cells during CAR T-cell therapy causes the CAR T-cells to lose their capacity to detect and eliminate tumor cells. To counteract these challenges and bolster the effectiveness of CAR T-cell therapy for patients with CD19-negative relapse, several innovative strategies have been introduced.

A particularly promising strategy involves shifting the targeted antigens of CAR T-cell therapy. In B cell lymphoma, CD22 expression is found in 91%–99% of both aggressive and indolent cases (72). Shah and colleagues highlighted that for relapsed/refractory patients with ALL who had prior treatment with CART19 cell therapy, utilizing CAR T-cells that targeted CD22 led to a 70% CR rate (73). Similarly, CD20, a defining feature of B cells, is being considered as a potential therapeutic target. In the context of B-cell lymphoma, alternative targets for CAR T-cell therapies are similarly being investigated, particularly for patients who have experienced a CD19-negative relapse after CART19 cell therapy. A phase I trial studying CD20-targeted CAR therapy involved patients with relapsed/refractory B-NHL who did not respond to CART19 cell therapy. This study reported an ORR of 100% at 7.8 months, with 71% of patients achieving CR. However, the incidence of CRS was more frequent than with CART19 cell therapy; all participants experienced some degree of CRS, with 85% being grade 1–2 (74). Recent findings on CD22-targeted CAR therapy for patients with CART19-refractory LBCL are also promising, demonstrating a 100% CR rate. At a median follow-up of 7.8 months (range: 6 to 9.3 months), all patients continued to be in CR. Similar to the CD20-targeted CART study, the rate of CRS was higher than with CART19 cell therapy, with all patients experiencing CRS (95% grade 1–2). Notably, in this study, 24% of patients developed macrophage-activation syndrome, characterized by pancytopenia and widespread intravascular coagulation (75).

In summary, when patients undergo CD19-negative relapse subsequent to CART19 cell therapy, the pursuit of alternative antigen targets such as CD20 and CD22 using CAR T-cell therapies represents a promising strategy to surmount resistance and enhance treatment outcomes. Nevertheless, additional research and comprehensive clinical trials are needed to substantiate the safety and efficacy of these modalities within a more expansive patient cohort.

### Dual- and tri-targeted CAR T-cell therapy

The development of dual-specific CAR T-cells, featuring combinations such as CD19/CD20, CD19/CD22, or CD20/CD22, and tri-specific CD19/CD20/CD22 CAR T-cells, is gaining momentum as a strategy to address antigen-negative relapse observed following single-targeted CART19 cell therapies (76). Strategies to target multiple distinct antigens simultaneously can be achieved through several methods: utilizing a bicistronic vector that enables the expression of two separate CARs within the same T cell, adopting co-transduction which encodes two CAR transgene constructs through multiple vector transduction, or employing tandem transduction that integrates two CARs within a singular chimeric protein via a single vector. Technological advancements have made dual- and tri-specific CART cell therapies more feasible, yet three primary challenges persist: (1) dual- or tri-specific CART cell therapy does not address resistance mechanisms that occur beyond the loss of target antigens; (2) there is a lack of comprehensive data of the safety and effectiveness of dual- or tri-specific CART cells *in vivo*; and (3) the larger transgene and payload size complicates the manufacturing process. Specific manufacturing hurdles for dual- and tri-specific CART cells include a lengthy optimization process to find suitable vectors, higher batch-to-batch variability in the production of viral vectors, lower transduction efficiency, and higher likelihood of manufacturing failures due to the complexities of the larger bicistronic vectors (63). Despite these challenges, the field of dual- and tri-specific CART cells has gained attention. These combined targeting approaches aim to address the variability in antigen densities present on tumor cells. By targeting multiple antigens simultaneously, the potential for tumor evasion due to antigen loss is greatly reduced. This strategy has the potential to extend therapeutic impact and yield more consistent responses, considering the natural variability found in malignant cell populations. For instance, Zhou et al. demonstrated preclinical efficacy of tri-specific CAR T-cells which used variable domains of heavy-chain antibodies (VHH) targeting CD19/CD20/CD22 in a lymphoma xenograft model. They observed improved proliferation, persistence, and tumor elimination *in vivo*, suggesting a potential treatment option for patients who relapse with CD19-negative or -dim variants (77). Spiegel and colleagues carried out a phase I clinical study on dual-targeting CD19/CD22 CAR T-cells in patients with relapsed/refractory B-ALL and LBCL (NCT03233854). All patients with LBCL were CAR-naïve, but in patients with B-ALL, 65% had previous CD19-directed therapy (including one patient who received previous CART19 cell therapy) and 29% had previous CD22-directed therapy. The study highlighted safety and significant effectiveness in B-ALL cases. The 6-month PFS for LBCL (29%, 95% CI 12%–48%) was on par with the real-world data for tisa-cel (35). However, a setback was observed as resistance to the dual-targeting CAR led to a relapse in patients with both CD19- and CD22-negative cells. They concluded that there is a need for more research to refine multi-targeting strategies in CAR T-cell therapies, aiming to enhance its effectiveness in B cell malignancies (78).

### Allogeneic HSCT following CAR T-cell therapy

Allogeneic HSCT is also considered a potential treatment option for relapse after CAR T-cell therapy and may offer a curative approach. The most extensively reported study on allogeneic HSCT following relapse after CART19 cell therapy for DLBCL is a retrospective analysis of 39 patients by Fried et al. (79). In this study, allogeneic HSCT was conducted a median of 127 days (range: 82–206 days) after CAR T-cell therapy. At the time of allogeneic HSCT, 21% of the patients had progressive disease. The 2-year OS and PFS were reported to be 45% and 31%, respectively. However, the 2-year cumulative incidence of non-relapse mortality (NRM) was 26%, with veno-occlusive disease/sinusoidal obstruction syndrome being the main adverse event, having a cumulative incidence of 15.4%. This highlights significant safety concerns with allogeneic HSCT.

Regarding the safety of allogeneic HSCT after CAR T-cell therapy, particularly concerning graft-vs.-host disease (GVHD), a retrospective analysis of patients with B-ALL has been reported (80). Compared to patients who received allogeneic HSCT after chemotherapy, patients who received allogeneic HSCT after CAR T-cell therapy showed higher incidence of Grade 2–4 acute GVHD (48.1% vs. 25.6%) and chronic GVHD (73.3% vs. 55.0%). However, the incidences of Grade 3–4 acute GVHD and extensive chronic GVHD were found to be similar. Even in cases targeting DLBCL, a comparably high occurrence of GVHD has been observed (79).

Limited data is available on allogeneic HSCT after relapse post-CAR T-cell therapy, and the establishment of optimal HSCT protocols in these scenarios is still ongoing. Various studies have demonstrated the effectiveness of reduced-intensity conditioning (RIC)-based allogeneic HSCT for treating NHL and Hodgkin lymphomas (81, 82). One report suggested superior outcomes with a fludarabine + melphalan-based conditioning regimen over a fludarabine + busulfan regimen for cord blood transplantation in lymphomas (83). A phase I/II trial for patients with ALL, NHL, or CLL who received allogeneic HSCT after CAR T-cell therapy reported an NRM rate of 15% for NHL, with a high NRM risk when myeloablative conditioning was used (84). Importantly, Corradini et al. highlighted the necessity of achieving and maintaining clinical and molecular remission before proceeding with RIC-based allogeneic HSCT (82). In recent studies, the efficacy of haploidentical HSCT (haplo-HSCT) using post-transplantation cyclophosphamide (PT-Cy) has been reported. A retrospective analysis using registry data from the European Society for Blood and Marrow Transplantation and Center for International Blood & Marrow Transplant Research from 2008 to 2015 for DLBCL displayed comparable transplant outcomes for PT-Cy-based haplo-HSCT using nonmyeloablative or RIC to HLA-matched donor transplantation with 3-year OS and NRM of 46% and 38%, respectively (85). In comparison to matched sibling donors and matched unrelated donors, there was a reduced cumulative incidence of chronic GVHD. As previously discussed, achieving and maintaining CR before allogeneic HSCT is paramount, but it remains a significant hurdle. Another study on salvage therapy for relapsed DLBCL post-CAR T, which included five allogeneic HSCT cases of a total of 120, revealed better outcomes for CAR T responders than for non-responders (86). However, outcomes based on rescue therapy choices were not significantly different; the responsiveness to rescue therapies was influenced by factors like prior treatment responsiveness to CAR T-cell therapy and elevated LDH at relapse. Without improving treatments as discussed in subsequent sections, better outcomes with allogeneic HSCT post-relapse treatment will remain elusive. Zurko et al. also retrospectively evaluated the results of allogeneic HSCT after CAR T failure in patients with LBCL. The median interventional therapies between CAR T administration and allogeneic HSCT was 1 (range: 0–7). RIC-based regimens were employed in 77% of cases, with peripheral blood as the graft source in 86% of instances. The donor classifications included 39% matched unrelated donors, 30% haploidentical donors, and 26% matched related donors. The median observational duration for survivors stood at 15 months (range: 1–72 months). The estimated one-year OS, PFS, and GVHD-free relapse-free survival rates were 59%, 45%, and 39%, respectively. The rates for one-year NRM and progression/relapse were 22% and 33%, respectively. Multivariate analysis demonstrated that fewer than 2 interventional therapies between CAR T and allogeneic HSCT, along with a CR at the time of allogeneic HSCT, were correlated with superior outcomes. Consequently, the authors deduced that allogeneic HSCT is a viable consideration for patients who attain CR after CAR T-cell therapy failure (87).

### Other therapies

Some studies have explored the therapeutic efficacy of alternative agents, though they predominantly encompass small patient cohorts or are based on individual case reports. Loncastuximab tesirine, a CD19-targeted therapy, received accelerated approval for treating adult patients with relapsed/refractory DLBCL, a decision influenced by the results of the phase II LOTIS-2 trial (88). In this trial, 13 patients with DLBCL who had relapsed after CAR T-cell therapy were treated with loncastuximab tesirine. Approximately 46% of these patients showed a response to the treatment. Notably, the median OS for these patients was 8.2 months, with a PFS of 1.4 months, and the duration of response was 8 months (89). Further emphasizing its potential, the global phase II study of loncastuximab monotherapy (NCT03075696) underscored the drug's efficacy and manageable safety profile in heavily pretreated DLBCL patients (90). Additionally, patients who underwent CAR T-cell therapy subsequent to loncastuximab treatment exhibited an ORR of approximately 40%–50% (91).

Additionally, Zhu Y, et al. reported a study focusing on the outcomes of venetoclax for patients with DLBCL post-CAR T-cell therapy, involving a cohort of 10 patients. The achieved ORR was 80%, and the CR was 30% (92). Further case studies have also cited the use of agents like BTKi, histone deacetylase inhibitors, and ibrutinib (93, 94).

## Conclusion

CAR T-cell therapy has revolutionized the therapeutic landscape for B cell malignancies, offering promising response rates, especially in DLBCL and ALL. CART19 cell therapy has demonstrated significant clinical responses, with many patients achieving CR. Despite the undeniable potential of CAR T-cell therapy, there remain pressing challenges. Most prominently, resistance or relapse post-treatment occurs in a significant proportion of patients. In response to the challenge of relapse, a plethora of strategies are under investigation (Table 4). The re-administration of CART19 cells, although showing some efficacy, is inherently limited due to potential CD19 mutations or losses. The therapeutic shift towards targeting alternative antigens, such as CD22 and CD20, is a more promising strategy. More ambitiously, dual- or tri-targeting CAR T-cells could potentially circumvent the limitations of single antigen reliance, ensuring that the evasion tactics of tumor cells are minimized. Beyond cellular therapies, several agents and treatments are being trialed for their efficacy in post-CAR T relapse scenarios. The effectiveness of lenalidomide, both as a salvage and potential maintenance therapy post-CAR T, stands out as particularly promising. RT, especially for localized lesions, and allogeneic HSCT offer viable treatment avenues, although each has its own set of challenges and considerations. Allogeneic HSCT presents risks such as GVHD that need to be weighed against its curative potential, but it should be highly considered when patients are in CR. Agents such as loncastuximab tesirine and venetoclax demonstrate potential efficacy, although the existing data regarding their effectiveness are still emerging. Consequently, larger-scale clinical trials are necessary to substantiate these findings and to further evaluate their therapeutic impact. Additionally, the appropriateness of salvage therapies for MCL and FL remains uncertain, with limited research available on the subject. There is a clear need for more comprehensive studies in this area.

**Table 4:** Summary of salvege therapies post-CAR T-cell therapy.

Therapy	Author	ORR (%)	CR (%)	1-year OS (%)	1-year PFS (%)
Second CART19 cell infusion	Gauthier	52	33	48	13
Immune checkpoint inhibitor	Tomas	20	20	NA	NA
	Major	19	10	30	13
Polatuzumab Vedotin	Tomas	48	52	37	NA
	Gouni	44	14	NA	NA
Lenalidomide	Tomas	33	33	69	NA
	Blasi	11	6.7	NA	NA
BTK inhibitor	Tomas	43	7	21	NA
Radiation	Tomas	54	27	NA	NA
	Blasi	36	14	NA	NA
	Imber	85	23	NA	NA
Allogenic HSCT	Fried	61	43	53	30
	Zurko	NA	NA	59	45

ORR, overall response rate; CR, complete response; OS, overall survival; PFS, progression-free survival; CART19, CD19-directed chimeric antigen receptor T cell; BTK, Bruton's tyrosine kinase; HSCT, hematopoietic stem cell transplantation; NA, not applicable.

Ultimately, the complexity of managing relapses post-CAR T-cell therapy underlines the necessity for continuous research. The dynamic interplay between CAR T-cells and malignant B-cells, especially the latter's capacity for adaptability and evolution, emphasizes the importance of developing multi-pronged therapeutic strategies. The ultimate goal remains to extend the therapeutic benefits of CAR T-cell therapy, ensuring durable responses, and most importantly, improving patient outcomes.
